# Repellency and toxicity of a CO_2_-derived cedarwood oil on hard tick species (Ixodidae)

**DOI:** 10.1007/s10493-022-00692-0

**Published:** 2022-01-25

**Authors:** Lina B. Flor-Weiler, Robert W. Behle, Fred J. Eller, Ephantus J. Muturi, Alejandro P. Rooney

**Affiliations:** 1 U.S. Department of Agriculture, Agricultural Research Service, National Center for Agricultural Utilization Research, Crop BioProtection Research Unit, 1815 N University St., Peoria, IL 61604 USA; 2 U.S. Department of Agriculture, Agricultural Research Service, National Center for Agricultural Research, Functional Foods Research Unit, 1815 N University St., Peoria, IL 61604 USA; 3 US Department of Agriculture, Agricultural Research Service, Cropping Systems Research Laboratory, 3810 Fourth St., Lubbock, TX 79415 USA

**Keywords:** Cedarwood oil, *Amblyomma americanum*, *Dermacentor variabilis*, *Ixodes scapularis*, *Rhipicephalus sanguineus*, Repellency, Toxicity

## Abstract

The repellency and toxicity of a CO_2_-derived cedarwood oil (CWO) was evaluated against actively questing unfed nymphs of four species of hard ticks: *Amblyomma americanum* (L.), *Dermacentor variabilis* (Say), *Ixodes scapularis* Say, and *Rhipicephalus sanguineus* (Latreille). Using a vertical climb bioassay for repellency, nymphs of these species avoided a CWO-treated filter paper in proportional responses to treatment concentrations. At 60 min of exposure, *I. scapularis* nymphs were most sensitive with 50% repellency concentration (RC_50_) of 19.8 µg cm^−2^, compared with RC_50_ of 30.8, 83.8 and 89.6 µg cm^−2^ for *R. sanguineus, D. variabilis* and *A. americanum*, respectively. Bioassays determined the lethal concentration for 50% (LC_50_) and 90% (LC_90_) mortality of nymphs exposed to CWO in treated vials after 24- and 48-h exposure. After 24 h exposure, the LC_50_ values were 1.25, 3.45 and 1.42 µg cm^−2^ and LC_90_ values were 2.39, 7.59 and 4.14 µg cm^−2^ for *D. variabilis*, *I. scapularis* and *R. sanguineus*, respectively, but had minimal effect on *A. americanum*. After 48 h exposure, the LC_50_ values were 4.14, 0.78, 0.79 and 0.52 µg cm^−2^, and LC_90_ values were 8.06, 1.48, 1.54 and 1.22 µg cm^−2^ for *A. americanum*, *D. variabilis*, *I. scapularis* and *R. sanguineus*, respectively. The repellency of CWO on tick species decreased with time. The repellency and toxicity bioassays demonstrated concentration-dependent responses of tick nymphs to the oil, indicating the potential of the CO_2_-derived cedarwood oil be developed as an eco-friendly repellent and/or acaricide.

## Introduction

Ticks are important ectoparasites that serve as reservoirs for a wide range of zoonotic pathogens (bacteria, viruses and protozoa). These obligate hematophagous arthropods rank second only to mosquitoes for their impact on humans and animals worldwide as vectors of infectious agents causing serious illnesses (Sonenshine et al. 2002; Benelli et al. 2016). Hard ticks (Ixodidae) are capable of harboring several medically important pathogens that can be transmitted synchronously to a susceptible host (Swanson et al. 2006; Reis et al. 2011). Over the last few decades, the USA has witnessed a steady rise in cases of notifiable tick-borne diseases with nearly 50,000 cases reported annually (CDC, 2019). This up-surge for human tick-borne illnesses may be a result of the expanding tick populations (Merten and Durden 2000; Jongejan and Uilenberg 2004; Hahn et al. 2018). Although tick habitats are associated with natural woodland, grassland or forested areas, these areas are also suitable habitats for host animals and frequently visited by humans for outdoor recreational activities. This combination provides for a favorable association between disease harboring ticks and susceptible humans, creating a substantial public health challenge.

Despite significant research efforts, development of vaccines to counter tick-borne diseases remains in its infancy (Merino et al. 2013; Hassan et al. 2019; Trentelman et al. 2019). Thus, disease prevention strategies rely primarily on managing tick-vector populations. Over the years, synthetic acaricides have been effectively used to control all stages of ticks. This approach has serious drawbacks as it contributes to environmental pollution that affects non-target organisms and incites pesticide resistance in tick populations (Sonenshine et al. 2006; Balbus et al. 2013; Abbas et al. 2014; Khater et al. 2016; Eiden et al. 2017; Rodriquez-Vivas et al. 2018).

These negative impacts of synthetic acaricides have prompted research for alternative control strategies for suppressing tick populations and vectored diseases. Plant compounds, especially essential oils, exhibit biological activity against arthropods and have been used to repel and kill ticks (Carroll et al. 2010, 2011, 2017; Flor et al. 2011; Tabanca et al. 2013; Hue et al. 2015; Benelli et al. 2016; Faraone et al. 2019; Salman et al. 2020) as well as reduce tick reproduction, oviposition and egg viability (Divya et al. 2014; Shyma et al. 2014). Repellents act as barriers for personal protection against tick bites thus prevent transmission of tick-borne diseases. Chemical constituents of essential oils such as nootkatone and carvacrol effectively repelled *Ixodes scapularis* Say with 50% repellent concentration (RC_50_) values of 0.05 and 0.07%, respectively (Panella et al. 2005; Deitrich et al. 2006). Citronellol and geraniol at 0.103 mg cm^−2^ repelled > 90% of *Amblyomma americanum* (L.) nymphs (Hsouna and Hamdi 2012), whereas juniper (*Juniperus communis*), palmarosa (*Cymbopogon martini*), cedar (*Cedrus atlantica*), lemon grass (*Cymbopogon citratus*), ginger (*Zingeber officinale*), geranium (*Pelargonium graveolens*) and bergamot (*Citrus aurantium var bergamia*) oils at 1, 5 and 10% oil concentrations affected reproduction and reduced oviposition and egg hatchability by *Rhipicephalus microplus* (Canestrini) ticks (Pazinato et al. 2016). Furthermore, nootkatone from grapefruit was toxic to *A. americanum*, *Dermacentor variabilis* (Say), *I. scapularis* and *Rhipicephalus sanguineus* (Latreille) (Flor-Weiler et al. 2011).

Cedarwood oil (CWO) from *Calocedrus decurrens* is toxic to *I. scapularis* ticks (Dolan et al. 2007) and CWO from Eastern red cedar (ERC), *Juniperus virginiana*, is a known repellent to several species of ants (Eller et al. 2014, 2015). In addition, CWO prevents both termites and wood-decay fungi from attacking otherwise susceptible wood (Eller et al. 2010, 2018, 2020). The most common method for essential oil extraction from cedarwood is steam distillation, a process that has several disadvantages including low oil yield and altered oil characteristics (Eller et al. 2018). An alternative method using CO_2_ extraction produced the highest yield of cedarwood oil with higher levels of cedrol, which is reported to be one of the most bioactive components of CWO (Eller et al. 2018). Hence, we evaluated the repellency and toxicity of the CO_2_-extracted CWO against questing nymphs of four species of hard ticks.

## Materials and methods

Unfed nymphs (1–2 days since molting) of four tick species (*A. americanum*, *D. variabilis*, *I. scapularis* and *R. sanguineus*) were procured from the Tick Rearing Facility, National Tick Research and Education Resource (at the Department of Entomology and Plant Pathology, Oklahoma State University, Stillwater, OK, USA). Nymphs of each species were held separately in 15-ml (= 4-dram) vials (Fisherbrand, Vineland, NJ, USA) capped with cotton fabric that was secured with a rubber band and covered with aluminum foil. Vials with ticks were stored in a glass desiccator with saturated solution of potassium sulfate in water (97%) to maintain high relative humidity (90–95%). Desiccators were placed in an incubator (Percival Scientific, Perry, IA, USA) at 21 °C and L10:D14 photoperiod. Prior to bioassays, nymphs were acclimated to ambient laboratory conditions for at least 24 h.

### Source of cedarwood oil

*Juniperus virginiana* heartwood sawdust was prepared from a locally harvested tree (Woodford County, IL, USA) as previously described by Eller et al. (2014) and the CWO was extracted from the sawdust using supercritical CO_2_ (70 °C, 27.6 MPa) (Eller and King 2000). Gas chromatography (GC) was used to determine the CWO composition as described by Eller and Taylor (2004) and the GC analysis indicated that the six most abundant components (relative peak area) which accounted for nearly 90% of the components in the CWO were: α-cedrene (14%), β-cedrene (4%), thujopsene (19%), cuparene (4%), cedrol (40%) and widdrol (8%).

### Repellency bioassay

**Fig. 1:**
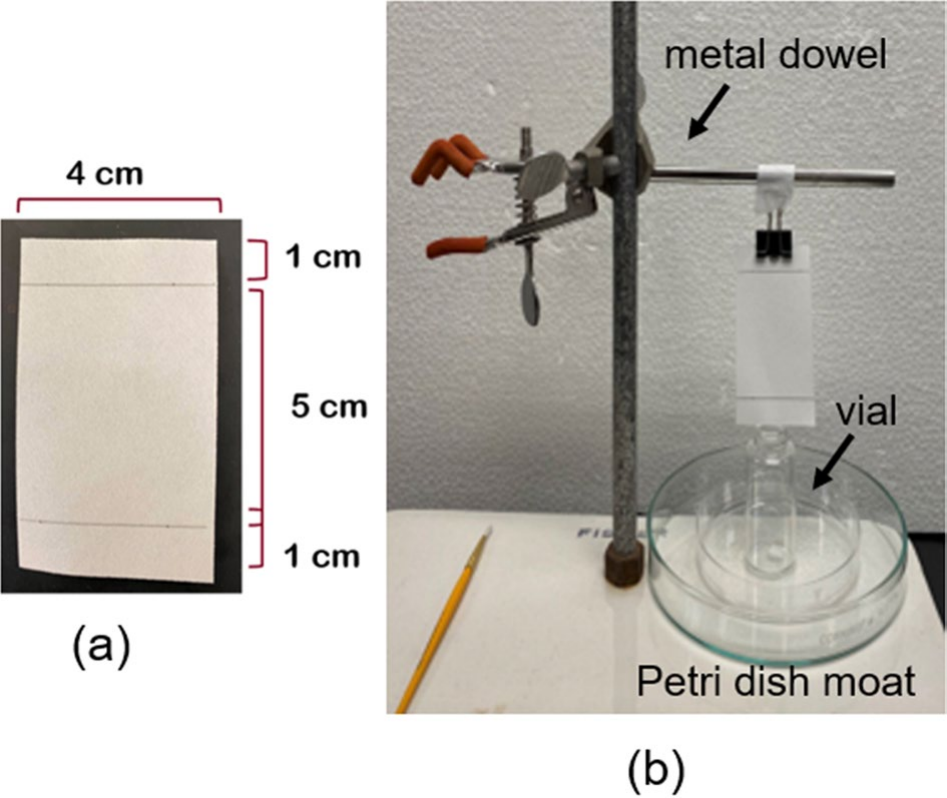
Repellency bioassay. **a** A marked filter paper strip (7 × 4 cm, Whatman no. 4) creating three zones for CO_2_-derived cedarwood oil (CWO) concentration treatment. **b** Set-up with treated filter paper strip suspended over a Petri dish moat, hanging vertically from a slender metal dowel using a small binder clip to hold one of the untreated zones of the filter paper strip. Tick nymphs are transferred into the vial placed at the center of the Petri dish moat with the rim of the vial touching the lower untreated zone of the filter paper strip, allowing the nymphs to crawl onto the filter paper strip

Tick host-seeking behavior is observed as the propensity to climb vertical surfaces. The climbing behavior is exploited by this experiment designed to measure repellency. Repellency was based on observations of vertical climbing over treated substrate as modified from Carroll et al. (2004). A 7 × 4 cm rectangle filter paper (Whatman no. 4) was marked with a pencil to create three zones; a 4 × 1 cm zone at each end of the paper leaving a 5 × 4 cm zone (20 cm^2^) in the middle (Fig. 1a). For treatment, the marked filter paper was placed in a glass Petri dish (15 mm high, 100 mm diameter; Corning, Glendale, AZ, USA) and the middle zone was evenly treated with 165 µL of CWO diluted in hexane (see below) using a pipettor. The treated filter paper strip was hung vertically in a fume hood using a small, binder clip (Skilcraft, New Britain, CT, USA) for 15–20 min for the hexane to evaporate before each test. The treated paper strips were then transferred and hung vertically from a slender metal dowel using the binder clip to hold one of the untreated zones. The treated paper strips were suspended over a moat consisting of a Falcon polystyrene Petri dish (15 mm high, 100 mm diameter) glued centrally to a larger Petri dish (15 mm high, 150 mm diameter; Corning Life Sciences, Durham, NC, USA). The larger outer dish was filled with water to contain ticks that dropped from the filter paper (Fig. 1b).

Four concentrations of CO_2_-derived CWO and two control treatments (six sample treatments) were tested to compare repellency of CWO to nymphs of the four tick species. Cedarwood oil was serial diluted (2 ×) in hexane from 16 to 1 mg mL^−1^ for application of 165 µL per dilution to filter papers. The resulting treatment rates of 132, 66, 33 and 16.5 µg cm^−2^ CWO for exposure of larger *A. americanum* and *D. variabilis* nymphs, and 66, 33, 16.5 and 8.25 µg cm^−2^ CWO for exposure of smaller *I. scapularis* and *R. sanguineus* nymphs. Determined concentrations were based on preliminary tests conducted. Higher concentrations were tested for *A. americanum* and *D. variabilis* based on preliminary tests that showed the need for higher concentrations to better bracket a 50% response. The negative control treatment was hexane only and the positive control was DEET (N,N-diethyl-meta-toluamide, 98.11% Repel; United Industries, St. Louis, MO, USA) at 32 µg cm^−2^. The concentration of DEET used as the standard positive control (32 µg cm^−2^) was based on preliminary test that repelled 100% of test tick species, determined through a dose–response test conducted using four concentrations of DEET. DEET was serial diluted (2 ×) in hexane from 4 to 0.5 mg mL^−1^ resulting in treatment rates of 32, 16, 8 and 4 µg cm^−2^ and tested for repellency to nymphs of the four tick species. Only naive nymphs were used in bioassays. The treatments and controls were tested sequentially in a randomized order in each of the replicate experiments.

Nymphs were kept in a desiccator with saturated potassium sulfate solution in water (97%) to maintain high RH storage conditions until use in bioassays. Naive nymphs were then immobilized by placing storage vials in an ice bath. Then, 60–70 nymphs were transferred into a clean uncovered 15-ml (4-dram) vial using a fine paint brush. This vial with nymphs was placed in the center of the plastic Petri dish moat, held beneath the treated filter paper strip such that nymphs could crawl onto the lower untreated zone of the filter paper. Ten nymphs were allowed to crawl onto the filter paper or were transferred from the rim of the vial to the untreated portion of the filter paper using a fine paint brush. The locations of the nymphs were recorded at 10, 30 and 60 min after the 10th tick climbed or was placed onto the filter paper. Repellency assays were conducted at daytime (10:00–17:00 h) under fluorescent lights at 20–21 °C temperature and 60–75% RH. The observer was at least 30–45 cm from the treated vertical paper strip to record tick locations.

Ticks were considered repelled if they remained on the lower untreated part of the strip or if they dropped off the strip without having crossed into the upper untreated zone. Ticks that crawled onto the upper untreated zone or the clip were removed to prevent their return to the lower untreated zone. Each treatment sample was assayed 5 × for replication and providing evaluation of 50 exposed nymphs, whereas for the DEET dose–response test, each treatment sample was assayed 3 × for replication, providing evaluation of 30 exposed nymphs.

### Coating of vials and toxicological bioassay

Four serial dilutions (5 ×) of CWO were made with hexane ranging from 0.5 to 0.004 mg mL^−1^. These dilutions (1 mL) were used to coat the inner surface of 15-ml (4-dram) vials (63.16 cm^2^ inner surface) with CWO at concentrations of 7.92, 1.58, 0.32 and 0.06 µg cm^−2^ following the method by Flor-Weiler et al. (2011). Coating of vials were done by adding 1 mL of dilute CWO treatment into each vial using a pipet. Vials with dilute CWO were gently shaken and placed on their side on a roller (Bellco Biotechnology, Vineland, NJ, USA) in a fume hood for 20–30 min or until hexane had completely evaporated, leaving the CWO. Then, 10 nymphs were introduced into each treated vial and the vials were capped with a piece of cotton fabric secured with a rubber band and covered with aluminum foil. Vials treated with hexane-only were included as the negative control. Each toxicological assay consisted of three replicate vials (10 nymphs per vial) per treatment and the assay was repeated 3 ×, thus 90 nymphs were exposed to each CWO concentration and the control treatment for each tick species tested. Nymphs were used only once in bioassays. Tick mortality was recorded 24 and 48 h after exposure to treated vials. Ticks were considered moribund or dead if they were incapable of movement, failed to maintain normal posture, exhibited uncoordinated movement, were unable to right themselves, or remained motionless when prodded.

### Statistical analysis

The concentration repellency response data from the vertical filter paper bioassay and dosage-response acaricidal results obtained in toxicity bioassay were evaluated by probit analysis (Finney 1971) using SAS v.9.4 statistical package (SAS Institute, Cary, NC, USA) to calculate concentrations for 50 and 90% repellency (RC_50_ and RC_90_) and the lethal concentrations for 50 and 90% morbidity (LC_50_ and LC_90_), along with 95% confidence intervals. Since calculations of lethal dose ratios were not provided in SAS statistical software, differences between tick species exposed to CWO concentrations for repellency and toxicity were designated as significant if the 95% confidence interval (CI) of their respective RC_50_ (repellency) or LC_50_ (toxicity) did not overlap. Furthermore, repellency data among tick species at different concentrations over time were subsequently analyzed by ANOVA generated by SAS v.9.4. Prior to analysis, repellency data (%) were arcsine√x-transformed to meet the assumptions of normality. Differences on mean percent repellency among tick species to CWO were separated using a Tukey’s honestly significant difference (HSD) test (α = 0.05).

## Results

CWO repelled nymphs of *A. americanum*, *D. variabilis*, *I. scapularis* and *R. sanguineus* compared with the no-oil control treatment. Results of probit analysis showing the effective concentration (RC_50_ and RC_90_) values for repellency are summarized in Table 1. The negative control (hexane only solvent) did not elicit a repellent response among the nymphs tested (0% repellency). Upon crawling into the treated filter paper, hard tick nymphs did not move fast and remained longer in the untreated portion of the filter paper before attempting to climb upward and calculated values for repellency increased with exposure time. Thus, we consider the 60-min evaluation to best represent the ability of the treatments to repel the ticks. Among the species tested, CWO showed greater repellency against *I. scapularis* and *R. sanguineus* nymphs with lower RC_50_ values at the 10-, 30- and 60-min evaluation (Table 1). *Ixodes scapularis* and *R. sanguineus* were more sensitive to the oil with RC_50_ and RC_90_ values statistically lower when compared with RC values for *A. americanum* and *D. variabilis* at 30- and 60-min evaluations. The RC_50_ values for *I. scapularis* and *R. sanguineus* were not statistically different from each other but were statistically different from *A. americanum* and *D. variabilis* based on non-overlapping 95% confidence interval (Table 1). Although we used a positive control DEET at 32 µg cm^−2^ as our standard positive control that provided 100% repellency against all tick species, the dose–response test for DEET tested against nymphs required lower concentrations to repel nymphs of the four hard tick species. The RC_50_ and RC_90_ values for DEET were not statistically different among species over time, and all demonstrated a clear-concentration repellency response with the concentrations tested (Table 2). By comparison, a higher concentration of CWO was needed to repel tick nymphs based on the RC_50_ and RC_90_ values except for *I. scapularis* nymphs that had a comparable RC_50_ value with DEET, 19.8 µg cm^−2^ CWO to 19.1 µg cm^−2^ DEET at 60-min evaluation (Tables 1 and 2).

**Table 1:** Repellent concentration (RC_50_ and RC_90_) values for repellency of CO_2_-derived cedarwood oil (CWO) against nymphal stages of four tick species after 10, 30 and 60 min exposure

Exposure	Species	CWO µg cm^−2^ (95% confidence interval)		Equation
		RC_50_	RC_90_	
10 min	*Amblyomma americanum*	64.4 (48.3–81.8)c	173.4 (140.3–241.3)c	*y* = 0.012*x* − 0.758
	*Dermacentor variabilis*	43.9 (17.2–62.9)c	179.7 (138.6–283.1)c	*y* = 0.009*x* − 0.413
	*Ixodes scapularis*	3.2 (-8.7–9.2)a	31.0 (25.2–42.6)a	*y* = 0.046*x* − 0.146
	*Rhipicephalus sanguineus*	17.1 (10.3–22.5)b	52.1 (43.9–66.3)b	*y* = 0.037*x* − 0.625
30 min	*A. americanum*	76.5 (63.3–92.8)b	167.3 (139.1–216.9)c	*y* = 0.014*x* − 1.081
	*D. variabilis*	51.7 (32.2–68.9)b	169.4 (134.5–246.4)c	*y* = 0.011*x* − 0.564
	*I. scapularis*	10.3 (3.5–14.8)a	35.8 (29.9–46.9)a	*y* = 0.050*x* − 0.519
	*R. sanguineus*	20.0 (13.1–25.8)a	58.6 (49.4–74.7)b	*y* = 0.033*x* − 0.666
60 min	*A. americanum*	89.6 (77.4–105.1)b	165.9 (142.5–205.1)b	*y* = 0.017*x* − 1.503
	*D. variabilis*	83.8 (68.2–105.2)b	191.3 (155.2–264.6)b	*y* = 0.012*x* − 0.999
	*I. scapularis*	19.8 (12.4–25.8)a	60.1 (50.3–77.4)a	*y* = 0.032*x* − 0.629
	*R. sanguineus*	30.8 (24.4–37.3)a	72.5 (61.2–92.2)a	*y* = 0.031*x* − 0.944

Concentrations for repellency within a column and within an exposure period followed by different letters are significantly different (based on no overlap between 95% confidence intervals)

**Table 2:** Repellent concentration (RC_50_ and RC_90_) values for repellency of DEET (positive control) against nymphal stages of four tick species after 10, 30 and 60 min exposure

Exposure	Species	DEET µg cm^−2^ (95% confidence interval)		Equation
		RC_50_	RC_90_	
10 min	*Amblyomma americanum*	18.8 (15.9–22.8)	32.4 (27.4–41.2)	*y* = 0.094*x* − 1.794
	*Dermacentor variabilis*	15.2 (12.7–18.6)	27.4 (23.1–35.4)	*y* = 0.105*x* − 1.607
	*Ixodes scapularis*	11.5 (8.2–14.7)	26.6 (21.7–36.7)	*y* = 0.084*x* − 0.970
	*Rhipicephalus sanguineus*	13.6 (11.2–16.6)	24.4 (20.4–31.8)	*y* = 0.119*x* − 1.611
30 min	*A. americanum*	20.2 (17.5–23.8)	30.9 (26.8–37.8)	*y* = 0.120*x* − 2.420
	*D. variabilis*	18.5 (16.0–21.7)	27.7 (24.0–34.2)	*y* = 0.138*x* − 2.549
	*I. scapularis*	15.2 (12.4–18.6)	28.7 (23.9–37.4)	*y* = 0.095*x* − 1.441
	*R. sanguineus*	18.3 (15.8–21.7)	28.4 (24.5–35.2)	*y* = 0.126*x* − 2.325
60 min	*A. americanum*	21.2 (18.7–24.5)	29.2 (25.7–34.9)	*y* = 0.161*x* − 3.428
	*D. variabilis*	20.8 (18.3–24.1)	28.8 (25.3–34.7)	*y* = 0.160*x* − 3.334
	*I. scapularis*	19.1 (16.3–22.7)	30.7 (26.3–38.2)	*y* = 0.110*x* − 2.093
	*R. sanguineus*	20.1 (17.6–23.4)	28.7 (25.1–34.8)	*y* = 0.149*x* − 2.910

Concentrations for repellency within a column and within an exposure period were not significantly different (based on overlap between 95% confidence intervals)

**Fig. 2:**
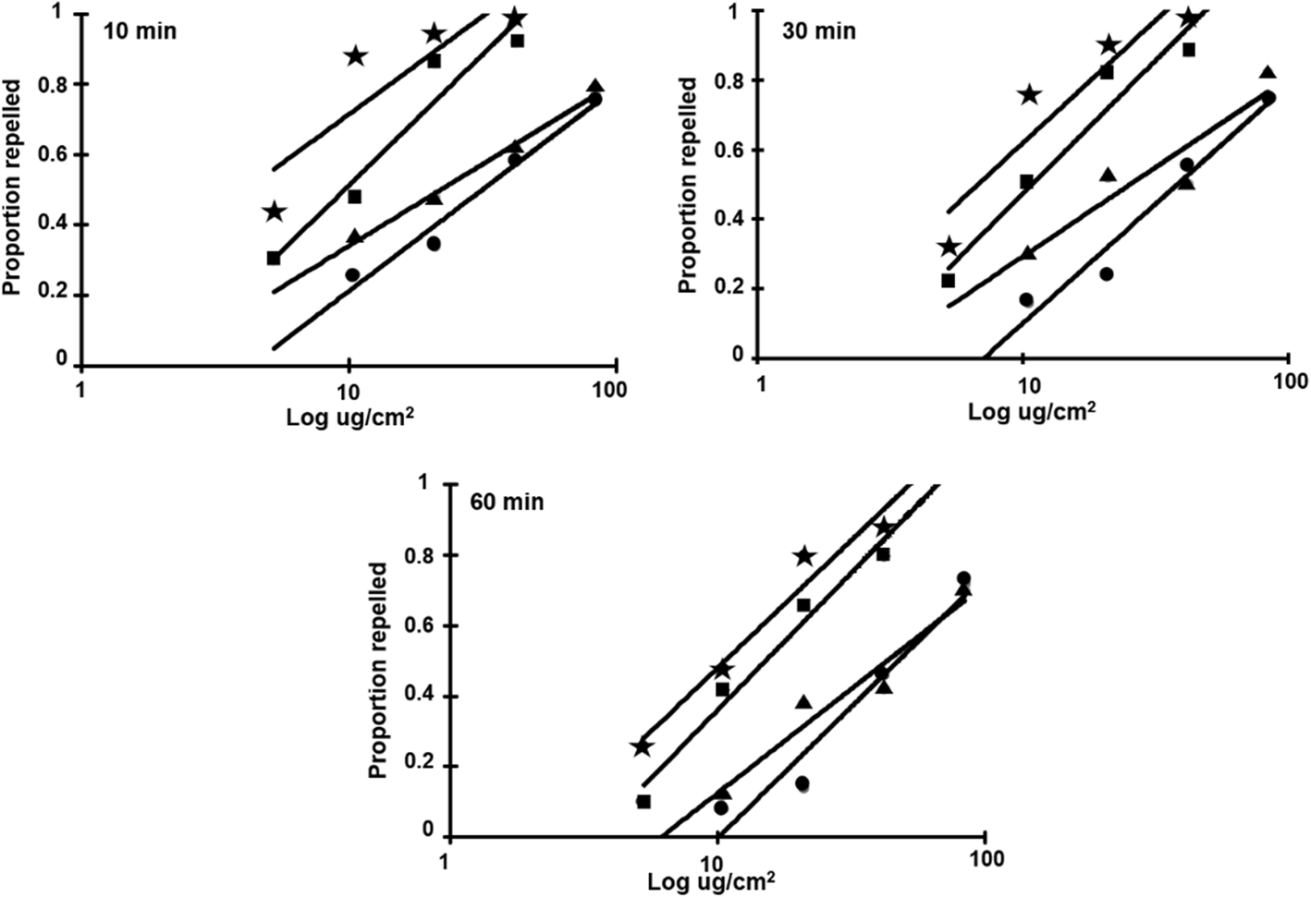
Responses of hard tick nymphs to various concentrations of CO_2_-derived cedarwood oil (CWO) applied in vertical filter paper assays. Five replications of 10 ticks were tested over time. *Amblyomma americanum* (filled circle), *Dermacentor variabilis* (filled triangle), *Ixodes scapularis* (filled star) and *Rhipicephalus sanguineus* (filled rectangle)

For all four tick species tested, repellency increased with increasing oil concentration consistent with a dose–response relationship and *I. scapularis* was most sensitive to CWO. Figure 2 illustrates the proportion of nymphs repelled relative to log concentration treatments of CWO. For each time point, *I. scapularis* nymphs were the most sensitive, followed by *R. sanguineus*, *D. variabilis*, then *A. americanum*. Repellency of CWO against *I. scapularis* nymphs was 94% (*F*_5,29_ = 33.41), 90% (*F*_5,29_ = 83.37) and 80% (*F*_5,29_ = 58.43, all *P* < 0.0001) at 10-, 30- and 60-min exposure to 33 µg cm^−2^ CWO, respectively, which is significantly higher than repellency against *A. americanum* and *D. variabilis*. After 60-min exposure, the higher concentrations of CWO remained effective. The treatment concentration at 132 µg cm^−2^ CWO repelled 72 and 70% of *A. americanum* and *D. variabilis* nymphs, respectively. CWO at 66 µg cm^−2^ concentration repelled 80 and 88% of *R. sanguineus* and *I. scapularis*, respectively, a repellency that is significantly higher against *A. americanum* and *D. variabilis* with only 46 and 42% repellency, respectively (*F*_5,29_ = 109.52, *P* < 0.0001). Repellency of hard tick nymphs to CWO decreased over time. Tick repellency among nymphs over 30- to 60-min exposure to CWO at 33 µg cm^−2^ was reduced by 11–41%. Repellency reduction among species was 41, 27, 19 and 11% for *A. americanum*, *D. variabilis*, *R. sanguineus* and *I. scapularis*, respectively, whereas the high concentration of CWO for *A. americanum* and *D. variabilis* (132 µg cm^−2^) reduced repellency by 3 and 14%, respectively, over 30- to 60-min exposure. The high concentration of CWO for *I. scapularis* and *R. sanguineus* (66 µg cm^−2^) reduced nymph repellency by 10 and 14%, respectively, over 30- to 60-min exposure.

The toxicity assay showed all four tick species to be susceptible to CWO except that *A. americanum* nymphs did not exhibit morbidity characteristics at the 24-h evaluation (Table 3). For the other three species evaluated at 24 h, LC_50_ values were 1.25, 3.45 and 1.42 µg cm^−2^ and the LC_90_ values were 2.39, 7.59 and 4.14 µg cm^−2^ for *D. variabilis, I. scapularis* and *R. sanguineus*, respectively. CWO was significantly more toxic to *D. variabilis* and *R. sanguineus* compared with *I. scapularis*. There was no mortality recorded for *A. americanum* at 24 h of exposure and thus no LC values for comparison. At 48 h of exposure, *A. americanum* had significantly higher LC_50_ and LC_90_ values for CWO exposure than the other three tick species (Table 3). Nymphs of *A. americanum* were less susceptible to CWO toxicity compared with nymphs of the other three tick species.

**Table 3:** Probit analysis for mortality of tick nymphs at 24 and 48 h of continuous exposure to CO_2_-derived cedarwood oil (CWO)

Exposure (h)	Species	CWO µg cm^−2^ (95% confidence interval)		Equation
		LC_50_	LC_90_	
24	*Amblyomma americanum*	–	–	–
	*Dermacentor variabilis*	1.25 (1.08–1.46)a	2.39 (2.06–2.89)a	*y* = 1.123*x* − 1.404
	*Ixodes scapularis*	3.45 (2.93–4.06)b	7.59 (6.65–8.88)c	*y* = 0.310*x* − 1.070
	*Rhipicephalus sanguineus*	1.42 (1.08–1.86)a	4.14 (3.35–5.50)b	*y* = 0.472*x* − 0.672
48	*A. americanum*	4.14 (3.60–4.76)c	8.06 (7.15–9.27)b	*y* = 0.327*x* − 1.353
	*D. variabilis*	0.78 (0.67–0.90)a	1.48 (1.31–1.70)a	*y* = 1.843*x* − 1.442
	*I. scapularis*	0.79 (0.67–0.91)a	1.54 (1.36–1.78)a	*y* = 1.698*x* − 1.337
	*R. sanguineus*	0.52 (0.42–0.63)b	1.22 (1.03–1.47)a	*y* = 1.848*x* − 0.967

Concentrations for toxicity within a column and within an exposure period followed by different letters are significantly different (based on no overlap between 95% confidence intervals)

## Discussion

Several studies have been published looking onto various methods for evaluating the efficacy of repellents against ticks (Dautel 2004; Carroll et al. 2004, 2011, 2016; Dietrich et al. 2006; Bissinger et al. 2016; Lima Ada et al. 2016; El-Seedi et al. 2017; Elmhalli et al. 2019; Mawella et al. 2019). Different repellency bioassay methods have been used including horizontal or Petri dish bioassay (Carroll et al. 2004; Faraone et al. 2019), open filter bioassay (Elmhalli et al. 2019), impregnated and non-impregnated filter paper on vertical glass rod bioassay (Lima Ada et al. 2016) and vertical bioassay (Carroll et al. 2004; Meng et al. 2016). The vertical repellency method used in this study exploits the tick’s host-seeking behavior to climb vertical surfaces, making the experiment simple, effective and easy to observe (Carroll et al. 2004). Bioassay results demonstrated different responses to CWO among the four hard tick species. Differences in response to repellents among tick species have been reported previously (Carroll et al. 2004; Dietrich et al. 2006; Meng et al. 2016; Ferreira et al. 2017; Faraone et al. 2019). In our study, nymphs of *I. scapularis* and *R. sanguineus* were more sensitive to the oil where *I. scapularis* was strongly repelled with RC_50_ values of 3.2, 10.3 and 19.8 µg cm^−2^ at 10-, 30- and 60-min exposure (Table 1). When *I. scapularis* nymphs were exposed to CWO concentration of 33 µg cm^−2^, the oil repelled 90 and 80% nymphs at 30- and 60-min exposure, respectively (Fig. 2). In contrast, *A. americanum* required higher concentrations of CWO for repellency with RC_50_ values of 64.4, 76.5 and 89.6 µg cm^−2^ at 10-, 30- and 60-min exposure, respectively. The CWO concentration of 33 µg cm^−2^ only repelled 24 and 14% *A. americanum* nymphs at 30- and 60-min exposure. Unfed nymphs of *A. americanum* were less sensitive to the oil compared to *D. variabilis*, *I. scapularis* and *R. sanguineus*. Our results mirrored those reported by Carroll et al. (2004) where *A. americanum* required higher concentrations of the AI13-37220 compound for repellency when compared with *I. scapularis*. Similarly, higher concentrations of essential oil extracts were required for repellency of *D. variabilis* compared with concentrations required to repel *I. scapularis* (Carroll et al. 2016).

The repellent activity may vary depending on the size of ticks. *Amblyomma americanum* nymphs (1.5–2.5 mm) are known to be longer than nymphs of the other species tested: *I. scapularis* (0.9–1.3 mm), *D. variabilis* (0.9 mm) and *R. sanguineus* (0.5 mm) (Keirans et al. 1996; NCIPMI 1998). However, Deitrich (2006) expounded that the effectiveness of a compound to repel ticks depends on multiple characteristics, including the test medium (i.e., skin vs. filter paper), age, physical state of the tick, and the presence or absence of host-associated stimuli. It is important to note that in the case of *D. variabilis*, it is the adult stage that bites dogs and humans and based on our results, higher concentrations of repellency would likely be needed against the large, fast-moving adults.

Our data showed that CWO actively repelled hard tick nymphs. The dose–response test for DEET exhibited a comparable RC_50_ value with CWO against *I. scapularis* at 60-min evaluation with RC_50_ values of 19.8 and 19.1 µg cm^−2^ for CWO and DEET, respectively. This demonstrates that among hard tick nymphs tested, the repellency of CWO can be as effective as DEET on *I. scapularis* nymphs. For the other hard tick species tested, higher CWO concentrations are needed compared to DEET. We have also observed that tick repellency to CWO concentrations decreased over time. It has been documented that essential oil components are volatile, and their volatility is linked to reduction of its effectiveness (Regnault-Roger et al. 2012). The volatility of CWO may be one of the reasons for the decrease in repellency over time among ticks. Volatility of components of essential oils can also be affected by the types and structure of test surface. For example, Amer and Mehlhorn (2006) reported that *Eucalyptus radiata* oil repelled 100% of *Culex quinquefasciatus* for 8 h when applied to human skin. Likewise, components of various essential oils applied to leaves of bean and cabbage exhibited significant repellent effect to two-spotted spider mites (Tak and Isman, 2017).

CWO was previously shown to be toxic to *I. scapularis* (Dolan et al. 2007), but CWO derived by high pressure CO_2_ extraction has not been extensively tested to evaluate toxicity among other hard tick species. Carbon dioxide extraction method by far gave the highest yield of high-quality CWO compared with other CWO extraction methods (Eller and King 2000). Essential oils contain several bioactive components that can be dominated by two or more substances. In the case of the CO_2_ extracted CWO, six most abundant components were identified (α-cedrene, β-cedrene, thujopsene, cuparene, cedrol and widdrol) and analysis has shown that it contains 3 × more cedrol and correspondingly lower α-cedrene where cedrol/α-cedrene ratio is an indicative quality of the oil. Cedrol is reported to be one of the most bioactive components of CWO (Eller and Taylor 2004; Eller et al. 2018). Although isolated cedrol is a solid at room temperature with a relatively high melting point (86–87 °C; Merck Index, 1989), it is part of a stable solution within the CWO mixture (Eller et al. 2015). Cedrol was reported to be one of the most termicidal components isolated from CWO (McDaniel et al., 1989; McDaniel and Dunn 1994) and cedrol alone was repellent to both red imported fire ants and little fire ants (Eller et al. 2014, 2015). Mun and Prewitt (2011) and Wang et al. (2011) both reported cedrol as one of the most active antifungal (wood-decay) components in extracts from *J. virginiana* and *Cunninghamia lanceolata*, respectively. Although cedrol is the most abundant component of the CO_2_-derived CWO and possibly the most active component as well, unfractionated CWO might be the most cost-effective material to use. Cedrol was toxic to *I. scapularis* with > 60% mortality observed at 0.063 mg mL^−1^ 24 h post exposure (Eller et al. 2014). Although we did not test the toxicity of cedrol on nymphs of other tick species, this compound may have mainly contributed to the toxicity of the oil on ticks. Weldon et al. (2011) correlated high repellency with high toxicity to ticks. Using cedrol for tick toxicity would be interesting to explore and fully realize the potential of the oil in effectively killing ticks.

The dose–response data in this study provided evidence that the CWO repels and is toxic to nymphs of all four hard tick species. The mode of action of CWO is based primarily on the repellent properties of the sesquiterpenes particularly cedrene and cedrol (US EPA, 1983) but the toxic effect needs to be further explored. The CWO sesquiterpenes were not found to have fumigation effects against termites when tested in closed containers, whereas the cedar needle terpenes killed 100% of the termites under identical conditions (Eller et al. 2010). The data presented in our study suggest that this natural product is toxic to ticks. Many essential oils are known to exhibit repellency and have insecticidal/acaricidal effect on various economically important arthropods (Isman 2000). Reports of Panella (1997) have demonstrated the acaricidal activity of essential oils against *I. scapularis* ticks and found the Alaska yellow cedar oil to be effective against nymphs (0.15% wt:vol) whereas the Eastern red cedar oil was most effective against larvae (0.001% wt:vol). Panizato et al. (2016) observed that cedar (*Cedrus atalantica*) oil inhibited egg hatchability and interfered with the reproduction of cattle ticks. One limitation to point out in our toxicity study is the lack of positive control using synthetic acaricide. However, studies using natural products and synthetic acaricides have been done. For example, Tabari et al. (2017) examined the toxicity and repellency of thymol, carvacrol and linalool from essential oil against *I. ricinus* and their observations revealed that carvacrol and thymol at 1, 2 and 5% killed 100% of *I. ricinus* larvae after 24 h, a larvicidal efficacy higher than that of permethrin with only 3, 7 and 23% larval mortality at 1, 2 and 5% concentration, respectively. Permethrin susceptibility of *I. scapularis* was also investigated by Burtis et al. (2021), exposing *I. scapularis* from three different sites (Shelter Island, Milbrook, NY, and CDC laboratory colony) to different concentrations of permethrin. *Ixodes scapularis* from CDC, Milbrook and Shelter Island after 24-h exposure to permethrin had LC_50_ values of 0.162, 0.244 and 0.303 mg mL^−1^, respectively.

Although the mode of action of CWO needs to be further understood, these findings indicate that CWO holds promise for the development of an effective eco-friendly repellent and/or acaricide. Further studies on the route of acaricidal action of the oil will provide practical information to determine appropriate formulations and effective delivery system that can be adopted for improved acaricidal potency and stability. Cedarwood oil (Virginia; CAS 8000–27-9) is listed as an active ingredient that can be used in pesticide products that are exempt from the Federal Insecticide, Fungicide, and Rodenticide Act (FIFRA) under the Minimum Risk Exemption regulations in 40 CFR 152.25(f) (United States Environmental Protection Agency under Active Ingredients Eligible for Minimum Risk Pesticide Products, updated December 2015). Cedarwood oil has also been designated ‘Generally Recognized as Safe’ (GRAS) by the US Food & Drug Administration (Code of Federal Regulations, Title 21, Chapter I, Subchapter B, Part 172, Subpart F, Section 172.515). Although further exploration for this oil is needed, CWO is perceived to be safe and environmentally acceptable.
